# The CD4+ T-cell transcriptome and serum IgE in asthma: IL17RB and the role of sex

**DOI:** 10.1186/1471-2466-11-17

**Published:** 2011-04-07

**Authors:** Gary M Hunninghake, Jen-hwa Chu, Sunita S Sharma, Michael H Cho, Blanca E Himes, Angela J Rogers, Amy Murphy, Vincent J Carey, Benjamin A Raby

**Affiliations:** 1 Channing Laboratory, Brigham and Women's Hospital, Harvard Medical School, Boston, MA 02115, USA; 2 Division of Pulmonary and Critical Care Medicine, Brigham and Women's Hospital, Boston, MA 02115, USA; 3 Center for Genomic Medicine, Brigham and Women's Hospital, Boston, MA 02115, USA; 4 Harvard-MIT Division of Health Sciences and Technology, Cambridge, MA, USA

## Abstract

**Background:**

The relationships between total serum IgE levels and gene expression patterns in peripheral blood CD4+ T cells (in all subjects and within each sex specifically) are not known.

**Methods:**

Peripheral blood CD4+ T cells from 223 participants from the Childhood Asthma Management Program (CAMP) with simultaneous measurement of IgE. Total RNA was isolated, and expression profiles were generated with Illumina HumanRef8 v2 BeadChip arrays. Modeling of the relationship between genome-wide gene transcript levels and IgE levels was performed in all subjects, and stratified by sex.

**Results:**

Among all subjects, significant evidence for association between gene transcript abundance and IgE was identified for a single gene, the interleukin 17 receptor B (IL17RB), explaining 12% of the variance (r^2^) in IgE measurement (p value = 7 × 10^-7^, 9 × 10^-3 ^after adjustment for multiple testing). Sex stratified analyses revealed that the correlation between IL17RB and IgE was restricted to males only (r^2 ^= 0.19, p value = 8 × 10^-8^; test for sex-interaction p < 0.05). Significant correlation between gene transcript abundance and IgE level was not found in females. Additionally we demonstrated substantial sex-specific differences in IgE when considering multi-gene models, and in canonical pathway analyses of IgE level.

**Conclusions:**

Our results indicate that IL17RB may be the only gene expressed in CD4+ T cells whose transcript measurement is correlated with the variation in IgE level in asthmatics. These results provide further evidence sex may play a role in the genomic regulation of IgE.

## Background

Total serum immunoglobulin E (IgE) is a risk factor for both the development of [1] and disease severity in asthma [2]. The production of IgE is controlled by a complex regulatory process that ultimately involves isotype class switching by mononuclear B lymphocytes, [3] a CD4+ T cell dependent process [3]. However, to our knowledge, there has been no analysis of the correlation between genome-wide CD4+ T cell gene expression and the variability in serum IgE among asthmatics.

Sex is a critical determinant of IgE level, with males having a stronger tendency towards higher total IgE than females [4]. Sex-related differences in IgE are present early in life [5] and persist into adulthood [6]. Furthermore, there is growing evidence that the underlying molecular underpinnings of IgE levels differ between males and females. For example, we have previously demonstrated striking differences by sex in IgE heritability estimates, with 83% of the variability in IgE levels attributable to genetic factors in males, as compared to 63% in females [7]. Moreover, evidence for sexually-distinct genetic determinants of IgE has been demonstrated in genome-wide linkage analyses of IgE in some, [7] but not all studies [8].

Based on these findings we hypothesized that genome-wide CD4+ T cell microarray analyses would provide unique insights into genes that correlate with the variability in IgE level, and that sex would be an important modifier of these correlations. To test these hypotheses we explored the relationship between total serum IgE levels and genome-wide gene expression transcript abundance in a cohort of 223 young adults with asthma, both in all subjects and within each sex specifically.

## Methods

### Study population and sample collection

CAMP was a multicenter clinical trial of the effects of anti-inflammatory medications in children with mild to moderate asthma. All participants had asthma defined by symptoms greater than twice per week, use of an inhaled bronchodilator at least twice weekly or use of daily medication for asthma, and increased airway responsiveness [9]. Participant data relating to atopy was obtained from the original CAMP dataset. The completion of the trial was followed by two four-year observation studies - CAMP Continuation Study (CAMPCS) 1 and 2. During Years 3 and 4 of CAMPCS/2, 299 children at four of eight CAMP study centers (Baltimore, Boston, Denver, St. Louis) participated in an ancillary study of gene expression in asthma. The study visit included questionnaire assessments of asthma severity, measurements of pulmonary function, and collection of peripheral blood for the simultaneous measurement of cell count, total serum IgE, and CD4+ T cell gene expression. Samples for RNA extraction were collected with BD Vacutainer CPT tubes, (BD Diagnostics, Franklin Lakes, New Jersey) placed on ice, centrifuged within 1 hour (20 minutes at 1700RCF) followed by mononuclear cell layer isolation and suspension in 10 ml of PBS. We isolated CD4+ T cells using anti-CD4+ microbeads by column separation (Miltenyi Biotec, Auburn, CA), [10] using 20 ul anti-CD4+ Micro beads per 10^6 ^total cells. Pilot studies confirmed CD4+ cell yields of ~4 × 10^6 ^at ≥ 95% purity per collection. Total RNA was extracted using the RNeasy Mini Protocol (QIAGEN, Valencia, CA) [11] and stored at -80°C. 2100 Bioanayzer (Agilent Technologies, Santa Clara, CA) analysis confirmed average total RNA yields of 2 ug per collection, with minimal evidence of RNA degradation and 28S:16S ratios approaching 2.0. Written informed consent was obtained from participants. CAMP was approved by the Institutional Review Boards of Brigham and Women's Hospital and the other participating centers.

### Microarray hybridization and sample preprocessing

Expression profiles were generated with Illumina HumanRef8 v2 BeadChip arrays (Illumina, San Diego CA) using 100 ng of CD4+ total RNA from each sample and the Illumina BeadStation 500G according to protocol [12]. Arrays were read using the BeadArray scanner (Illumina) and analyzed using BeadStudio (version 3.1.7) without background correction. Raw expression intensities were processed using the *lumi *package [13] with background adjustment with RMA convolution [14] and log_2 _transformation of each array. The combined set of arrays was subsequently quantile normalized. The Gene Expression Omnibus number for this dataset is GSE22324.

### Quantitative Real-Time PCR

For validation of our microarray results using real-time PCR (RT-PCR) was performed on a subset of subjects (n = 10) with the Taqman 7900 gene expression assay using primers and probes from Applied Biosystems. Quantitative RT-PCR analyses were performed in triplicate with appropriate positive and negative controls. The mRNA values were normalized with TATA binding protein (TBP) serving as the internal control. Relative mRNA expression levels were calculated using the delta cycle time (Ct) method.

### Serum Total IgE

Total serum IgE was measured by radioimmunosorbent assays from blood samples collected during the CAMPCS/2 visits. Serum IgE was transformed to the log_10 _scale for analysis.

### Pulmonary Function and Methacholine challenge testing

Pre- and post-bronchodilator spirometry was performed according to American Thoracic Society recommendations with a volume-displaced spirometer, and airway responsiveness was assessed by methacholine challenge with the Wright nebulizer tidal breathing technique [15].

### Statistical analysis

All expression microarray analyses were performed in R 2.9/Bioconductor [16,17]. Variance-based non-specific filtering was used to remove uninformative probes specifying a minimum interquartile range of 1 on the log_2 _scale. We used the *Limma *package [18] to analyze the linear correlation between gene expression level and log_10_IgE measurements both unadjusted for covariates and adjusted for age, sex (where appropriate), ethnicity, and center of sample collection. Joint and sex-stratified analyses were performed to assess the influence of sex. To control for multiple testing, we used the FDR procedure as proposed by Benjamini and Hochberg [19] with a threshold of 0.05 to define statistical significance. To generate multivariate linear models accounting for the large-*p *(expression transcript levels) small-*n *(number of subjects) problem, and to accommodate correlations among predictor variables, we used the least absolute shrinkage and selection operator (LASSO) [20] estimation. LASSO is a coefficient shrinkage and variable selection method that achieves model sparsity by setting coefficients of many irrelevant transcripts to 0. The LASSO analysis was performed using the least angle regression (LARS) algorithm, utilizing 5-fold cross-validation, as implemented in the R package *lars*. [21] We performed Canonical Pathway Analyses using Ingenuity Pathway Analysis ([IPA], Ingenuity Systems^®^, http://www.ingenuity.com) software (utilizing gene transcripts attaining nominal levels of significance [p < 0.05] in all subjects and within males and females specifically).

## Results

Expression data from peripheral blood CD4+ T cells was measured in 299 asthmatics from CAMP; 223 of these (75%) also had a simultaneous measurement of total serum IgE. The baseline characteristics of the 223 CAMP subjects included in this analysis are presented in Table 1. There were no significant differences in any clinical variable between subjects with and without IgE measurement. We tested for evidence of linear association between gene transcript abundance in peripheral blood CD4+ T cells and total serum IgE. After non-specific gene filtering, 13,310 (64.5%) transcripts demonstrating variable expression across samples were considered for testing.

**Table 1:** Baseline characteristics of subjects from the Childhood Asthma Management Program (CAMP)

Variable	Median (interquartile range) or Count (%)		
	**All (n = 223)**	**Males (n = 134)**	**Females (n = 89)**

Age, years	20 (19-22)	20 (19-22)	21 (19-23)

Sex, female	89 (40%)	-	-

Ethnicity, white	168 (75%)	97 (72%)	71 (80%)
African-American	42 (18%)	26 (19%)	16 (18%)
Hispanic	13 (6%)	11 (8%)	2 (2%)

Total serum IgE (IU/ml)	356 (145-924)	480 (150-1172)	253 (133-597)

Atopy *†	200 (90%)	122 (91%)	78 (88%)

Allergen Specific Skin Tests *‡			
Dust Mites			
*Dermatophagoides farinae*	97 (44%)	61 (46%)	36 (40%)
Dermatophagoides pternohyssinus	92 (41%)	58 (43%)	34 (38%)
Cockroaches			
*Blattella germanica*	66 (30%)	41 (31%)	25 (28%)
*Periplaneta Americana*	56 (25%)	36 (27%)	20 (22%)

Atopic dermatitis *‡§	48 (22%)	72 (43%)	15 (31%)

Allergic rhinitis *‡§	92 (42%)	56 (43%)	33 (35%)

FEV1 (liters) ‡	3.65 (3.13-4.19)	4.04 (3.65-4.48)	3.13 (2.86-4.48)

FEV1/FVC (%) ‡	78 (72-83)	76 (71-82)	79 (75-84)

PC20 (mg/ml) ‡	2.49 (1.64-4.88)	3.01 (1.88-4.88)	1.95 (1.37-4.53)

Used Inhaled Corticosteroids ‡║	50 (25%)	24 (21%)	26 (31%)

Used Oral Prednisone ‡║	12 (6%)	1 (1%)	11 (13%)

* Data on these atopy variables was recorded at enrollment into the CAMP trial (~10-12 years prior to both the gene expression and total serum IgE measurement).

**† **Atopy defined as subjects with at least one positive allergen skin test.

‡ Data missing for skin test positive responses to *Blattella Germanica *in 1 subject, atopic dermatitis FEV1 and FEV1/FVC ratio in 9 subjects, for inhaled corticosteroids or oral prednisone in 23 subjects, and PC20 in 34 subjects.

§ Atopic dermatitis and allergic rhinitis were defined by physician diagnosis.

║ Refers to subjects who used these medications within 7 days of both the gene expression and total serum IgE measurement.

### All Subjects

### Single Gene Regressive Strategies

**Figure 1:**
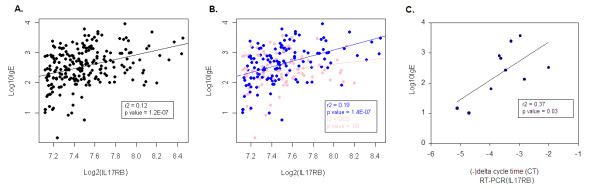
**The Correlation Between CD4+ Lymphocyte Measurement of IL17RB and Serum IgE**. Figure 1A and 1B represent plots of log_2_-transformed gene expression measurement of IL17RB (on the x-axis) vs. log_10_-transformed total serum IgE measurement (on the y-axis), with regression lines, for all subjects without (1A) and with (1B) consideration of male (in blue) and female (pink) sex. Figure 1C represents a plot of the (-) delta cycle time from the real time-polymerase chain reaction of measurement of IL17RB (on the x-axis) vs. log_10_-transformed total serum IgE measurement (on the y-axis) in all subjects.

Significant evidence for association between transcript abundance and total serum IgE was identified for a single gene, interleukin 17 receptor B (IL17RB), explaining 12% of the variance (r^2^) in total serum IgE measurement (p value = 7 × 10^-7^, 9 × 10^-3 ^after adjustment for multiple testing, see Figure 1A). Similar results were noted in analyses restricted to the 168 white subjects (IL17RB, p value = 2 × 10^-6^, 2 × 10^-2 ^after adjustment for multiple testing), and technical validation by RT-PCR confirmed the association between total serum IgE levels and IL17RB transcript abundance (a positive correlation between delta CT and total serum IgE [r^2 ^0.37, p value 0.03]). We re-analyzed our data with the inclusion of a quadratic term for gene expression level but found no significant findings of association under this non-linear modeling assumption. A list of the top 50 transcripts is presented in Additional File 1. No differences were noted in models additionally adjusting for measures of severity including methacholine PC_20 _(a measurement of airway responsiveness) or pre-bronchodilator FEV_1 _(a measurement of pulmonary function). Of note, exploratory analyses of the relationship between IL17RB transcript abundance and other intermediate asthma phenotypes revealed evidence of association with both total serum eosinophil count (p value = 6 × 10^-6^) and methacholine PC_20 _(p value = 4 × 10^-4^).

Due to the well-documented sexual dimorphism of IgE levels, we assessed whether sex would modify the association between gene expression and total serum IgE by repeating the analysis for males (n = 134, 60%) and females (n = 89, 40%) separately. These analyses revealed that the correlation between IL17RB and total serum IgE was restricted to males only (r^2 ^= 0.19, p value = 8 × 10^-8^, 1 × 10^-3 ^after adjustment for multiple testing), and was not observed in females (r^2 ^= .03, p value = 0.13, 1 after adjustment for multiple testing). Formal testing confirmed the observed differences in correlation of IL17RB and IgE in males and females was statistically significant (test for sex-specific interaction p < 0.05, see Figure 1B). It is important to note that the differences in correlation between IL17RB and IgE were not due to baseline differences in IL17RB levels between males and females (mean log_2 _IL17RB level 7.5 in both males and females, t-test p value 0.5 for the difference). No other transcript was associated with total serum IgE among either males or females after correction for multiple testing. Plots of the linear correlation between IL17RB level and total serum IgE in males and females specifically, are presented in Figure 1B. The gene transcript with the strongest correlation with total serum IgE among CAMP females (gamma-aminobutyric B receptor 1 [GABBR1], unadjusted p-value 9 × 10^-4^) did not remain significant after adjustment for multiple testing (p = 0.9998). Lists of the top 50 transcripts for both males and females are presented in Additional Files 2, 3.

Next we examined the degree of overlap in the correlations between gene transcript measurement and total serum IgE between males and females. At an arbitrary cutoff (p value < 0.05), there were 872 and 154 transcripts demonstrating correlation with total serum IgE among males and females, respectively. Only 16 of these 1010 unique transcripts (1.6%) were significantly correlated with total serum IgE in both males and females; and this result falls within the 95% confidence interval ([95% CI] 5-17) of the number of transcripts that should overlap by chance alone. At a more liberal threshold for inclusion of the results from females (p < 0.1), only 38 of 1205 unique transcripts (3.1%) overlap. At a more conservative threshold (p < 0.01) there were no transcripts common to both males and females.

We considered whether our findings of association in males but not females could be attributable to differences in sample size. Though power was somewhat lower in females due to the smaller number of subjects (89 females vs. 139 males), power was very high (>91%) to detect correlations half as strong as those observed among males (power 91.4% to detect an r^2 ^of 0.09 in the 89 CAMP females).

### LASSO

Next we evaluated whether an alternate multi-gene modeling approach utilizing 5-fold cross validation (the LASSO method) could both confirm our findings of association from single gene regressive strategies, and could identify additional determinants of total serum IgE levels. Among all subjects, the optimal multivariate model included 17 transcripts (including IL17RB), of which 16 map to unique HUGO gene identifiers (see Additional File 4). Consistent with our single gene regressive strategy the best fitting model includes *IL17RB*, the transcript with the largest estimated regression coefficient. Among males, the optimal multivariate model included 30 transcripts, including 6 that overlap with the best fitting regression model for all subjects: ASB9, H2A histone family, member J (H2AFJ), hexose-6-phosphate dehydrogenase (H6PD), IL17RB, poly(A) binding protein interacting protein 1 (PAIP1), and PVALB (see Additional File 5). Again, IL17RB was the transcript with the largest estimated regression coefficient among males. For females, LASSO selected the null model as its best prediction model, which suggests that none of the genes were informative in predicting IgE level.

### Ingenuity Canonical Pathway Analysis

**Figure 2:**
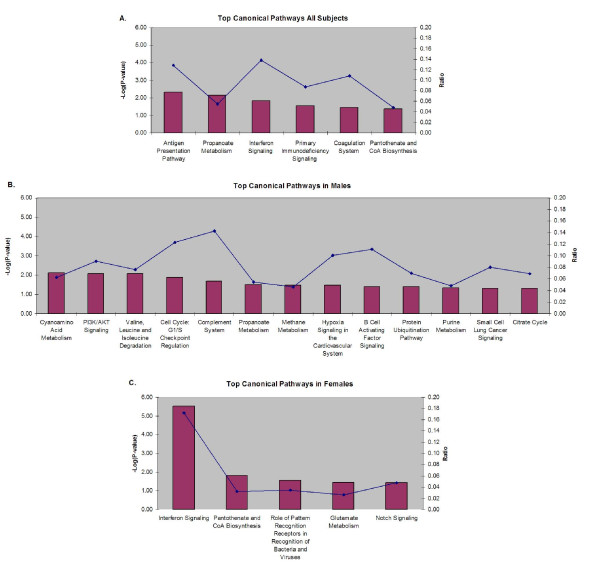
**Canonical Pathway Analysis of Serum IgE in CD4+ Lymphocytes**. Figures A-C represent the results of Canonical Pathway Analyses using Ingenuity Pathway Analysis ([IPA], Ingenuity Systems^®^, http://www.ingenuity.com) software in all subjects, males, and females respectively. The significance of the association between the our data set and identified pathways from the Ingenuity Pathway Analysis library of canonical pathways was measured as a ratio of the number of genes from our dataset that map to the pathway divided by the total number of molecules that exist within the canonical pathway (presented as a line plot). Fischer's exact tests were used to calculate p-values (presented as -log_10 _p values in a bar graph). In all subjects 763 transcripts demonstrated nominally significant (p < 0.05) correlation with total serum IgE. Of these 763 transcripts, 652 (85%) mapped to a unique HUGO gene id and were used for IPA canonical pathway analysis. Six pathways were nominally significant using a Fisher's exact test and are listed in Figure **2A**. Among males, 900 transcripts were correlated with total serum IgE, 873 (97%) mapped to a unique HUGO gene id, and thirteen pathways were significant (Figure **2B**). Among females, 158 transcripts were correlated with total serum IgE, 154 (97%) mapped to unique HUGO gene ids, and six pathways were significant (Figure** 2C**).

To better understand the combinatorial effect of multiple gene transcripts on IgE regulation we performed canonical pathway analyses. Of the 763 transcripts demonstrating nominally significant (p < 0.05) correlation with total serum IgE, 652 (85%) map to a unique HUGO gene id and were used for IPA canonical pathway analysis. Among all subjects, enrichment for six pathways was observed (Fisher's exact p < 0.05) including the antigen presentation pathway, propanoate metabolism, interferon signaling, primary immunodeficiency signaling, the coagulation system, and the pantothenate and CoA biosynthesis pathway (see Figure 2A). Similar to the regression models, sex-stratified pathway analysis revealed different patterns of pathway enrichment between males and females. Among males, nominal evidence for enrichment was observed for 13 pathways (Figure 2B). Among females, enrichment was observed for 6 pathways (Figure 2C), including strong evidence of enrichment for the interferon signaling pathway in females (p = 3.0 × 10^-6^). Notably, there were no significant canonical pathways in common between males and females. Additional information about the canonical pathway analyses, including lists of differentially expressed genes within the pathways are included in Additional Files 6, 7, 8. Of note, IL17RB is not currently a constituent of an Ingenuity canonical pathway.

## Discussion

This is the first study to correlate genome-wide CD4+ T cell transcript abundance with IgE level in humans and is, to our knowledge, the largest gene-expression study of as asthma-related quantitative phenotype published to date. Our results indicate that IL17RB may be the only gene expressed in CD4+ T cells whose transcript measurement is correlated with the variation in IgE level in asthmatics. Given that IgE level is strongly influenced by environmental factors (such as allergen exposures) it is surprising that 12% of the variation in IgE can be explained by IL17RB transcript measurement, a correlation that is substantially greater than that explained genetic determinants from three significant genomic regions (r^2 ^= 1.9%) identified in a recent genome-wide association study of IgE [22].

While it is often not possible within the confines of a cross-sectional epidemiologic study to determine the cause and effect relationship between two correlated variables, abundant molecular biologic evidence suggests that elevation of IL17RB should occur upstream from increases in IgE. IL17RB, located on chromosome 3p21.1, is the receptor for interleukin 17 B (IL17B) but has much higher binding avidity to interleukin 17 E ([IL17E] also known as IL25, IL-17Rh1, and Evi27 in the mouse) [23]. While structural homology places the IL17E-IL17RB ligand-receptor pathway within the IL17 family, as opposed to IL17A and IL17F which are cytokines characteristic of Th17 cells, [24] several groups have demonstrated that the IL17E-IL17RB pathway is a strong inducer of Th2 responses [25]. In humans, IL17RB expression has been demonstrated on the surface of lung fibroblasts, [26] prominently on activated CD4+ Th2 memory cells, [27] and recently in basophils [28]. Comparable to our findings, IL17RB has recently been shown to be the most differentially expressed gene in allergen challenged peripheral blood mononuclear cells from patients seasonal allergic rhinitis compared to healthy controls [28]. Thymic stromal lymphopoietin activated DCs appear to provide an important activating signal allowing IL17RB+ Th2 memory cells to respond to IL17E [27]. Elevated IL17E expression results in elevated Th2 cytokine production, [29] asthma severity, and elevations in IgE [30].

Another finding of our analyses is that the magnitude of the relationship between patterns of CD4+ lymphocyte gene expression and total serum IgE levels is substantially different between males and females. Several lines of evidence are provided, including: strong association of IL17RB transcript abundance with IgE levels in males (r^2 ^= 19%), but not in females (r^2 ^= 3%); evidence for a multivariate gene signature of total IgE levels in males, but not in females; and discrete, non-overlapping canonical gene pathway enrichment in each sex. Notably, we found limited statistical evidence to suggest that there is significant overlap between CD4+ T cell genes whose transcript measurements correlate with IgE level in both male and female asthmatics. Our work stands with a body of literature now implicating an important role for sex in the heritability, genome-wide linkage analyses, [7] and genome-wide gene expression analyses (^this report^) of IgE, and strongly support the notion that genetic and genomic studies of IgE should consider the role of sexual dimorphism.

Our study has several limitations. While our study is among the largest gene expression studies of asthma and allergy to date it is important to note that sample size may still be an important limitation of our analyses. The imbalance in sample size between males and females in this study, and the inherently lower mean values of IgE levels in females can further reduce statistical power. Though our power calculations suggest ample power (>90%) to detect the male-specific associations in our female sample, replication in larger sample sizes could help to determine if the lack of overlap we note between males and females is primarily an issue of the magnitude of the correlations or of true differences in the genomic regulation of IgE. In addition, our gene expression analyses include gene transcript measurements from unstimulated peripheral blood CD4+ lymphocytes in a population of adolescents and young adults recruited on the basis of childhood asthma. We urge caution in extrapolating our findings to other populations and cell types. Finally, while an assessment of IL17RB protein levels would provide additional supportive information, we do not have intact CD4+ lymphocytes enabling suitable cell surface protein quantification.

While the precise role that sex plays in the genomic regulation of IgE is unknown, sex differences in the immune environments experienced by CD4+ T cells are well described. In animals, females are more likely to develop a Th1 response after antigen challenge, except during pregnancy when a Th2 environment dominates [31]. Although little is known about the role sex could play in IL17RB pathway regulation, some evidence suggests that IL17RB level may influence the response to sex hormones [32]. We recently demonstrated sex-specific effects of *TSLP *polymorphisms on the regulation of specific, and total serum IgE in children with asthma [33]. This is intriguing given that TSLP activated dendritic cells (TSLP DCs) appear to be one of the most potent stimuli of IL17RB expression [27].

Though we have limited evidence that single genes, or genetic models strongly correlate with IgE level among females, it is intriguing that the canonical pathway most significantly correlated with IgE in females was the interferon signaling pathway (p = 3 × 10^-6^), particularly in light of prior studies suggesting the importance of interferon-γ (INFG) in the female (more than male) allergic response [34]. In mice, females demonstrate increased T-cell proliferative responses, [34] and consistently produce increased INFγ [35] in response to antigen challenge compared with their male counterparts. In humans, females have been shown to increase INFγ levels in response to a variety of stimuli [36]. Thus, our observation of differential down-regulation of the IFN signaling in females (but not in males) with higher levels of IgE is consistent with these prior studies and suggests an important regulatory role for interferon signaling in the female allergic response.

## Conclusions

Our results indicate that IL17RB may be the only gene expressed in CD4+ T cells whose transcript measurement is correlated with the variation in IgE level in asthmatics. We have demonstrated that sex is a critical modifier of the correlation between the CD4+ T cell transcriptome and IgE, and suggest that such sex-specific modification should be consideration in future studies of IgE.

## Competing interests

Dr. Raby reports receiving lecture fees from Novartis Pharmaceuticals. All of the other authors report no conflict of interest.

## Authors' contributions

GMH conducted the majority of the statistical analyses and drafted the manuscript. JHC, SSS, MHC, BEH, AJR, AM, participated in statistical analyses, and contributed to drafting this manuscript. VJC helped to create and modify the statistical packages used in these analyses, and contributed to drafting this manuscript. BAR conceived of the study, and participated in its design and coordination and helped to draft the manuscript. All authors read and approved the final manuscript.

## Pre-publication history

The pre-publication history for this paper can be accessed here:

http://www.biomedcentral.com/1471-2466/11/17/prepub

## Supplementary Material

Additional File 1 **Top 50 genes whose expression is correlated with total serum IgE in all subjects**. Click here for file

Additional File 2 **Top 50 genes whose expression is correlated with total serum IgE in males**. Click here for file

Additional File 3 **Top 50 genes whose expression is correlated with total serum IgE in females**. Click here for file

Additional File 4 **Genes included in Lasso model for all subjects**. Click here for file

Additional File 5 **Genes included in Lasso model for males. Additional File **3a: Click here for file

Additional File 6 **Canonical pathways (and genes) correlated with total serum IgE in all subjects**. Click here for file

Additional File 7 **Canonical pathways (and genes) correlated with total serum IgE in males**. Click here for file

Additional File 8 **Canonical pathways (and genes) correlated with total serum IgE in females**. Click here for file

## References

[B1] Burrows B Martinez FD Halonen M Barbee RA Cline MG Association of asthma with serum IgE levels and skin-test reactivity to allergens N Engl J Med 1989 320 271 277 10.1056/NEJM198902023200502 2911321

[B2] Sears MR Burrows B Flannery EM Herbison GP Hewitt CJ Holdaway MD Relation between airway responsiveness and serum IgE in children with asthma and in apparently normal children N Engl J Med 1991 325 1067 1071 10.1056/NEJM199110103251504 1891008

[B3] Geha RS Jabara HH Brodeur SR The regulation of immunoglobulin E class-switch recombination Nat Rev Immunol 2003 3 721 732 10.1038/nri1181 12949496

[B4] Guilbert TW Morgan WJ Zeiger RS Bacharier LB Boehmer SJ Krawiec M Larsen G Lemanske RF Liu A Mauger DT Atopic characteristics of children with recurrent wheezing at high risk for the development of childhood asthma J Allergy Clin Immunol 2004 114 1282 1287 10.1016/j.jaci.2004.09.020 15577824

[B5] Uekert SJ Akan G Evans MD Li Z Roberg K Tisler C Dasilva D Anderson E Gangnon R Allen DB Sex-related differences in immune development and the expression of atopy in early childhood J Allergy Clin Immunol 2006 118 1375 1381 10.1016/j.jaci.2006.09.008 17157669

[B6] Kerkhof M Droste JH de Monchy JG Schouten JP Rijcken B Distribution of total serum IgE and specific IgE to common aeroallergens by sex and age, and their relationship to each other in a random sample of the Dutch general population aged 20-70 years. Dutch ECRHS Group, European Community Respiratory Health Study Allergy 1996 51 770 776 8947333 10.1111/j.1398-9995.1996.tb00021.x

[B7] Raby BA Soto-Quiros ME Avila L Lake SL Murphy A Liang C Fournier E Spesny M Sylvia JS Verner A Sex-specific linkage to total serum immunoglobulin E in families of children with asthma in Costa Rica Hum Mol Genet 2007 16 243 253 10.1093/hmg/ddl447 17142250

[B8] Weiss LA Pan L Abney M Ober C The sex-specific genetic architecture of quantitative traits in humans Nat Genet 2006 38 218 222 10.1038/ng1726 16429159

[B9] The Childhood Asthma Management Program (CAMP): design, rationale, and methods. Childhood Asthma Management Program Research Group Control Clin Trials 1999 20 91 120 10.1016/S0197-2456(98)00044-0 10027502

[B10] Jonuleit H Schmitt E Schuler G Knop J Enk AH Induction of interleukin 10-producing, nonproliferating CD4(+) T cells with regulatory properties by repetitive stimulation with allogeneic immature human dendritic cells J Exp Med 2000 192 1213 1222 10.1084/jem.192.9.1213 11067871 PMC2193357

[B11] Gonzalez P Zigler JS Jr Epstein DL Borras T Identification and isolation of differentially expressed genes from very small tissue samples Biotechniques 1999 26 884 886 888-892 10337481 10.2144/99265st01

[B12] Barnes M Freudenberg J Thompson S Aronow B Pavlidis P Experimental comparison and cross-validation of the Affymetrix and Illumina gene expression analysis platforms Nucleic Acids Res 2005 33 5914 5923 10.1093/nar/gki890 16237126 PMC1258170

[B13] Du P Kibbe WA Lin SM lumi: a pipeline for processing Illumina microarray Bioinformatics 2008 24 1547 1548 10.1093/bioinformatics/btn224 18467348

[B14] Irizarry RA Hobbs B Collin F Beazer-Barclay YD Antonellis KJ Scherf U Speed TP Exploration, normalization, and summaries of high density oligonucleotide array probe level data Biostatistics 2003 4 249 264 10.1093/biostatistics/4.2.249 12925520

[B15] Smyth GK Bioinformatics and Computational Biology Solutions using R and Bioconductor 2005 New York: Springer

[B16] R Development Core Team R: A Language and Environment for Statistical Computing 2009 Vienna, Austria

[B17] Gentleman RC Carey VJ Bates DM Bolstad B Dettling M Dudoit S Ellis B Gautier L Ge Y Gentry J Bioconductor: open software development for computational biology and bioinformatics Genome Biol 2004 5 R80 10.1186/gb-2004-5-10-r80 15461798 PMC545600

[B18] Smyth GK Linear models and empirical bayes methods for assessing differential expression in microarray experiments Stat Appl Genet Mol Biol 2004 3 Article3 16646809 10.2202/1544-6115.1027

[B19] Benjamini Y Drai D Elmer G Kafkafi N Golani I Controlling the false discovery rate in behavior genetics research Behav Brain Res 2001 125 279 284 10.1016/S0166-4328(01)00297-2 11682119

[B20] Tibshirani R Regression shrinkage and selection via the lasso Journal of the Royal Statistical Society Series B 1995

[B21] Tibshirani R The lasso method for variable selection in the Cox model Stat Med 1997 16 385 395 10.1002/(SICI)1097-0258(19970228)16:4<385::AID-SIM380>3.0.CO;2-3 9044528

[B22] Weidinger S Gieger C Rodriguez E Baurecht H Mempel M Klopp N Gohlke H Wagenpfeil S Ollert M Ring J Genome-wide scan on total serum IgE levels identifies FCER1A as novel susceptibility locus PLoS Genet 2008 4 e1000166 10.1371/journal.pgen.1000166 18846228 PMC2565692

[B23] Tian E Sawyer JR Largaespada DA Jenkins NA Copeland NG Shaughnessy JD Jr Evi27 encodes a novel membrane protein with homology to the IL17 receptor Oncogene 2000 19 2098 2109 10.1038/sj.onc.1203577 10815801

[B24] Wang YH Liu YJ The IL-17 cytokine family and their role in allergic inflammation Curr Opin Immunol 2008 20 697 702 10.1016/j.coi.2008.09.004 18832032 PMC3702047

[B25] Angkasekwinai P Park H Wang YH Chang SH Corry DB Liu YJ Zhu Z Dong C Interleukin 25 promotes the initiation of proallergic type 2 responses J Exp Med 2007 204 1509 1517 10.1084/jem.20061675 17562814 PMC2118650

[B26] Lajoie-Kadoch S Joubert P Letuve S Halayko AJ Martin JG Soussi-Gounni A Hamid Q TNF-alpha and IFN-gamma inversely modulate expression of the IL-17E receptor in airway smooth muscle cells Am J Physiol Lung Cell Mol Physiol 2006 290 L1238 1246 10.1152/ajplung.00301.2005 16428271

[B27] Wang YH Angkasekwinai P Lu N Voo KS Arima K Hanabuchi S Hippe A Corrigan CJ Dong C Homey B IL-25 augments type 2 immune responses by enhancing the expansion and functions of TSLP-DC-activated Th2 memory cells J Exp Med 2007 204 1837 1847 10.1084/jem.20070406 17635955 PMC2118667

[B28] Wang H Mobini R Fang Y Barrenas F Zhang H Xiang Z Benson M Allergen challenge of peripheral blood mononuclear cells from patients with seasonal allergic rhinitis increases IL-17RB, which regulates basophil apoptosis and degranulation Clin Exp Allergy 40 1194 1202 10.1111/j.1365-2222.2010.03542.x 20545698

[B29] Fort MM Cheung J Yen D Li J Zurawski SM Lo S Menon S Clifford T Hunte B Lesley R IL-25 induces IL-4, IL-5, and IL-13 and Th2-associated pathologies in vivo Immunity 2001 15 985 995 10.1016/S1074-7613(01)00243-6 11754819

[B30] Ballantyne SJ Barlow JL Jolin HE Nath P Williams AS Chung KF Sturton G Wong SH McKenzie AN Blocking IL-25 prevents airway hyperresponsiveness in allergic asthma J Allergy Clin Immunol 2007 120 1324 1331 10.1016/j.jaci.2007.07.051 17889290

[B31] Whitacre CC Reingold SC O'Looney PA A gender gap in autoimmunity Science 1999 283 1277 1278 10.1126/science.283.5406.1277 10084932

[B32] Jerevall PL Brommesson S Strand C Gruvberger-Saal S Malmstrom P Nordenskjold B Wingren S Soderkvist P Ferno M Stal O Exploring the two-gene ratio in breast cancer--independent roles for HOXB13 and IL17BR in prediction of clinical outcome Breast Cancer Res Treat 2008 107 225 234 10.1007/s10549-007-9541-8 17453342

[B33] Hunninghake GM Lasky-Su J Soto-Quiros ME Avila L Liang C Lake SL Hudson TJ Spesny M Fournier E Sylvia JS Sex-stratified linkage analysis identifies a female-specific locus for IgE to cockroach in Costa Ricans Am J Respir Crit Care Med 2008 177 830 836 10.1164/rccm.200711-1697OC 18244952 PMC2292826

[B34] Weinstein Y Ran S Segal S Sex-associated differences in the regulation of immune responses controlled by the MHC of the mouse J Immunol 1984 132 656 661 6228595

[B35] Gourdy P Araujo LM Zhu R Garmy-Susini B Diem S Laurell H Leite-de-Moraes M Dy M Arnal JF Bayard F Herbelin A Relevance of sexual dimorphism to regulatory T cells: estradiol promotes IFN-gamma production by invariant natural killer T cells Blood 2005 105 2415 2420 10.1182/blood-2004-07-2819 15383462

[B36] Hewagama A Patel D Yarlagadda S Strickland FM Richardson BC Stronger inflammatory/cytotoxic T-cell response in women identified by microarray analysis Genes Immun 2009 10 509 516 10.1038/gene.2009.12 19279650 PMC2735332

